# T0001, a variant of TNFR2-Fc fusion protein, exhibits improved Fc effector functions through increased binding to membrane-bound TNFα

**DOI:** 10.1371/journal.pone.0177891

**Published:** 2017-05-19

**Authors:** Yijun Shen, Gang Li, Chunying Gu, Ben Chen, Aihua Chen, Hua Li, Bei Gao, Chencai Liang, Jingsong Wu, Tong Yang, Li Jin, Yong Su

**Affiliations:** 1 Ministry of Education Key Laboratory of Contemporary Anthropology, Fudan University, Shanghai, China; 2 R&D Department of Genetic Engineering, Shanghai Fudan-Zhangjiang Bio-Pharmaceutical Co., Ltd., Shanghai, China; Technische Universitat Munchen, GERMANY

## Abstract

T0001 is a recombinant human TNFR-Fc fusion protein mutant; it exhibits higher affinity to TNFα than etanercept and is now being tested in a Phase 1 study in China (ClinicalTrials.gov Identifier: NCT02481180). T0001 can inhibit the binding of soluble TNFα (sTNFα) or membrane-bound TNFα (mTNFα) to TNF receptors. When bound to mTNFα, the Fc-bearing TNFα antagonists have the potential to induce Fc-mediated effects, such as antibody-dependent cellular cytotoxicity (ADCC) and complement-mediated cytotoxicity (CDC) as well as outside-to-inside signals (apoptosis mainly). Recent studies have shown that ADCC may also play an important role in Crohn's disease (CD) and ulcerative colitis (UC). In this study, T0001 presented a higher binding activity on mTNFα than etanercept and similar binding activity with adalimumab and infliximab. Upon the addition of sTNFα, adalimumab and infliximab showed significantly increased binding to FcγRIIIa and C1q than T0001 and etanercept. T0001 exhibited significantly higher ADCC and CDC activity than etanercept, and the potency and the reporter response of T0001 were very close to adalimumab and infliximab in ADCC reporter gene assays. And the similar potency of T0001 was also corroborated by PMBC-based ADCC assay. T0001, but not etanercept could induce apoptosis, while adalimumab and infliximab were more effective. These results suggest that T0001 may not only exert improved efficacy in treating rheumatoid arthritis (RA) because of its high affinity to sTNFα but also has a therapeutic potential in CD and UC due to its increased binding to mTNFα with resultant Fc-associated functions (ADCC, in particular) and improved apoptosis.

## Introduction

Tumor necrosis factor α (TNFα) is a potent pro-inflammatory cytokine that exerts pleiotropic effects on various cell types and plays a critical role in the pathogenesis of chronic inflammation and autoimmunity diseases [1,2]. Two classes of TNFα antagonists are commercially available currently: soluble TNF receptor-Fc fusion protein (etanercept) and anti-TNFα monoclonal antibodies (mAbs) /fragments (adalimumab, infliximab, golimumab and certolizumab pegol); all five TNFα inhibitors are top sellers [3]. Recombinant human TNFR-Fc fusion protein mutant T0001 is a high affinity variant of etanercept, carrying a W89Y/E92N mutant in the TNFR domain. As we reported earlier, T0001 displays a 1.5-fold higher neutralizing activity and significant improvement in suppressing rat arthritis induced by collagen [4]. These data indicated that this high affinity variant may lead to improved efficacy in rheumatoid arthritis (RA) patients compared with etanercept. T0001 is now in phase 1 clinical trials to evaluate tolerance, pharmacokinetics and preliminary efficacy in patients with RA.

Therapeutic mAbs, including receptor-Fc fusion proteins, rely on two types of functionalities to achieve clinical efficacy: target-specific binding by the Fab or soluble receptor domain and immune-mediated effector functions by Fc domain. Antibody-dependent cellular cytotoxicity (ADCC) and complement-dependent cytotoxicity (CDC) are presumed to be key effector functions via interaction of the Fc domain with receptors on various cell types [5–8]. Although the binding and neutralizing activities against soluble TNFα (sTNFα) are the critical and common mechanisms of action (MOA) of these anti-TNFα agents, accumulating evidence suggests that not only sTNFα but also its precursor form, membrane-bound TNFα (mTNFα), are involved in the inflammatory response [9]. IgG1 antibodies targeting soluble ligands have low Fc effector function potential. However, if a membrane-bound form of the ligand points exists, the Fc effector function potential of the IgG1 therapeutic antibodies should be re-evaluated [10]. All TNFα antagonists can inhibit the binding of sTNFα or mTNFα to TNFR. When these agents bind to mTNFα, they have the potential to induce Fc-mediated effects, such as ADCC or CDC [11–13].

In RA, anti-TNFα mAbs are thought to act predominantly through the neutralization of sTNFα and mTNFα. In other conditions, such as Crohn’s disease (CD) and ulcerative colitis (UC), two main types of inflammatory bowel disease (IBD), signaling through a mTNFα and Fcγ receptor (triggering apoptosis or ADCC) may play a more important role [9,14,15]. In the present study, to explore the therapeutic potential of T0001 in CD and UC, we evaluated the binding characteristics, Fc effector functions and outside-to-inside signals (reverse signals) of T0001 compared with three representative clinically available anti-TNFα agents: etanercept, infliximab and adalimumab.

## Materials and methods

### Fusion protein and monoclonal antibodies

T0001 (carrying a W89Y/E92N mutant in the TNFR domain of etanercept) is not commercially available product, and it was prepared by Shanghai Fudan-Zhangjiang Bio-Pharmaceutical Co, Ltd. (China). The method to produce T0001 was fully described as a TNFR2-Fc variant (E92N/W89Y) by Tong Yang et al [4]. Infliximab (Remicade^®^), etanercept (Enbrel^®^), and adalimumab (Humira^®^) were purchased from Cilag AG, Pfizer, and AbbVie, respectively. Rituximab (Rituxan^®^, chimeric anti-human CD20 mAb), which was used as a control antibody, was purchased from Roche.

### The neutralizing assay on soluble TNFα-induced cytotoxicity in mouse L929 cells

Mouse L929 cells were seeded into 96-well tissue culture plates at a density of 1.5×10^4^ cells/well in DMEM (Basalmedia) supplemented with 3% FBS (Moregate). The cells were incubated at 37°C for 24 h and then incubated with gradient concentrations of TNFα antagonists in the presence of 1 μg/ml actinomycin D (Solarbio) and 20 IU/ml sTNFα (NIBSC, 12/154) for 18 h at 37°C. Cells without TNFα antagonists and sTNFα treatment served as controls. Cell viability was measured by MTS assay (Promega). In the MTS test, the cells in each well were incubated with MTS reagent mixture for 4 h, and MTS absorbencies were measured at 490 nm using a microplate reader SpectraMax M2^e^ (Molecular Devices). Percentage cell viability was calculated by dividing the absorbance values of drug treated wells by those of control wells and multiplying by 100. The dose response curve was fitted with a 4-parameter model using GraphPad Prism 5 software (GraphPad Software).

### Construction of membrane-bound TNFα-expressing cell lines

A membrane-bound TNFα (mTNFα) mutant resistant to TACE mediated cleavage, with amino acids 77 through 88 removed, was generated by site-directed mutagenesis as described previously [16]. The mTNFα DNA sequence was cloned into the pIRES vector (Clontech Laboratories, Inc.). The pIRES vector contained the CMV IE promoter driving expression of the mTNFα gene and a neomycin resistance marker for stable selection in mammalian cells. The transient transfected mTNFα cell line was created by transfection of mTNFα/pIRES in Jurkat T cells (ATCC, TIB-152) using Gene Pulser apparatus (Bio-Rad Laboratories, Hercules, CA). Two days after transfection, the cells were used in apoptosis assay. The stable mTNFα cell line was created by transfection of mTNFα/pIRES in CHO-K1 cells (ATCC, CCL-61). The transfected cells were selected by 500 μg/ml G418. Single-cell clones with high mTNFα expression were screened by a FACSCalibur flow cytometer (Becton Dickinson).

### Binding of TNF antagonists to mTNFα

T0001 was biotinylated using EZ-Link^®^ Sulfo-NHS-LC-Biotin (Thermo Scientific) according to the manufacturer’s recommendations. Varying concentrations of TNFα antagonists and 13.3 nM biotin-T0001 were co-incubated with 5×10^5^ mTNFα-expressing CHO cells or non-transfected CHO cells for 0.5 h at 4°C. The cells were washed with PBS to remove unbound agents and were then stained with R-phycoerythrin-conjugated avidin (Life Technologies) as a secondary antibody for 0.5 h at 4°C. Cells incubated with biotin-T0001 in the absence of drugs served as controls. Mean fluorescence intensities (MFI) were measured using a FACSCalibur flow cytometer. For data analysis, percentage competition was calculated as follows: percentage competition (%) = (MFI _control_ − MFI _drug_) / MFI _control_ ×100. The dose response curve was fitted with a 4-parameter model using GraphPad Prism 5 software.

### Construction of Jurkat-NFAT luciferase reporter cell line

The Jurkat-NFAT luciferase reporter cell line was constructed follow a previous description with some modifications [17]. Jurkat FcγRIII (158V) cells were created by co-transducing Jurkat T cells (ATCC, TIB-152) with the pcDNA3.1/hygro (Invitrogen) expressing the 158V allotype of human FcγRIIIa with a hygromycin resistance cassette and the pcDNA3.1/zeo (Invitrogen) vector expressing human FcεRIγ with a zeocin resistance cassette. Dual resistant colonies were screened by FACS for high FcγRIIIa expression and confirmed by anti-FcR cross linking induced IL-2 release. The reporter cell line Jurkat-NFAT luciferase was created by transducing Jurkat FcγRIII (158V) cells with the lentiviral vector-pGMLV-NFAT-Lu (Genomeditech) expressing luciferase reporter under the control of the NFAT promoter with a puromycin resistance cassette. Puromycin resistant colonies were screened by an NFAT activating agent (10 ng/ml PMA and 0.5 μM ionomycin), which induced luciferase expression with low non-induced background.

### Binding of TNF antagonists to FcγRIIIa

The non-TNFα target chimeric IgG1 mAb, rituximab was biotinylated using EZ-Link^®^ Sulfo-NHS-LC-Biotin according to the manufacturer’s recommendations. Varying concentrations of TNFα antagonists were incubated with or without 1:1 molar excess sTNFα (Sino Biological) for 1 h at room temperature, then were incubated with 2×10^5^ FcγRIIIa (158V) expressing Jurkat cells in the presence of 6.7 nM biotin-IgG1 for 0.5 h at 4°C. The cells were washed with PBS to remove unbound agents and stained with Streptavidin R-PE Conjugate (Life Technologies) as a secondary antibody for 0.5 h at 4°C. Cells incubated with biotin-IgG1 in the absence of drugs served as controls. MFI were measured using a FACSCalibur flow cytometer. For data analysis, percentage competition was calculated as follows: percentage competition (%) = (MFI _control_ − MFI _drug_) / MFI _control_ ×100. The dose-response curve was fitted with a 4-parameter model using GraphPad Prism 5 software.

### ADCC-reporter gene assay

The mTNFα expressing CHO cells were seeded into 96-well white opaque tissue culture plates at a density of 5×10^3^ cells/well in RPMI 1640 (Gibco). TNFα antagonists were serially diluted and incubated with the CHO cells for approximately 1 h at 37°C, 5% CO_2_. Following incubation, Jurkat-NFAT luciferase reporter cells were added to the CHO/antagonists mixture at 1×10^5^ cells per well. The mixture was incubated for approximately 6 h at 37°C, 5% CO_2_, and then the raw relative luminescence units (RLUs) were measured using a Bio-Glo^™^ Luciferase Assay system (Promega). For data analysis, a fold of induction (FI) was calculated as follows: FI = (RLU_induced_ − RLU_background_) / (RLU_uninduced_ − RLU_background_) [18]. The dose response curve was fitted with a 4-parameter model using GraphPad Prism 5 software. Rituximab was also tested against WIL2-S (CD20+) cells as a system positive control and tested against mTNFα-expressing CHO cells as a system negative control.

### Classical ADCC assay

Classical ADCC assay was performed using PBMCs as effector cells. Briefly, The mTNFα expressing CHO cells were seeded into 96-well flat, clear bottom plates at a density of 1×10^4^ cells/well in RPMI 1640 supplemented with 5% low IgG FBS (PAN) followed by addition of serial dilutions of TNFα antagonists. PBMCs (HemaCare) were then added to assay plates, at a density of 2×10^5^ cells per well. After 4 h incubation at 37°C, 5% CO_2_, target cell lysis was measured by detecting the release of lactate dehydrogenase (LDH) using Cytotoxicity LDH Assay Kit-WST (Dojindo) according to the manufacturer's instructions. The plates were read on a SpectraMax M2^e^ (Molecular Devices) plate reader for absorbance at 490 nm. Spontaneous LDH release was measured in wells containing target and effector cells without antibody. Maximal LDH release was measured in wells containing target cells completely lysed by lysis buffer supplied in the assay kit. For data analysis, the extent of specific ADCC was calculated as follows: % lysis = (experimental LDH release − spontaneous LDH release) / (maximal LDH release − spontaneous LDH release) × 100.

### Binding of TNF antagonists to C1q

We coated 96-well plates (Nunc) with 5 μg/mL goat anti-human C1q antibody (Accurate Chemical & Scientific Corporation) for 16 h at 4°C and blocked with PBST/3% BSA for 2 h at room temperature; a 100 μL/well of 3 μg/mL C1q (Quidel Corporation) was added to each well and incubated for 2 h at room temperature. Varying concentrations of TNFα antagonists and 133.3 nM biotin-rituximab were added to the plates alone or in the presence of a 0.8-fold molar excess sTNFα. Plates were incubated at room temperature for 15 h. The streptavidin-HRP (Beyotime) was added to the plates for 1 h at room temperature. Plates were washed with PBS containing 0.05% Tween-20 after each incubation step. Peroxidase activity was detected with TMB reagent (Thermo Scientific), and the plates were incubated at room temperature for 0.5 h to allow color development. The reaction was terminated with 1 M H_2_SO_4_, and the absorbance was measured at 450 nm (the background measured at 650 nm was subtracted for each well) using a SpectraMax M2^e^ microplate reader. Cells incubated with biotin-IgG1 in the absence of drugs served as controls. For data analysis, percentage competition was calculated as follows: percentage competition (%) = (Value _control_ − Value _drug_) / Value _control_ ×100.

### CDC assays

The mTNFα-expressing CHO cells were seeded into 96-well black plates (CORNING) at a density of 5×10^4^ cells/well in GMEM medium. TNFα antagonists were serially diluted and incubated with the CHO cells for approximately 1 h at 4°C. The cells and TNFα antagonists were then incubated in the presence of human complement serum (Quidel) to a final concentration of 8%, for 2 h at 37°C, 5% CO_2_. At the end of incubation, 50 μl of AlamarBlue^™^ (Invitrogen) was added to each well, and the plate was incubated for an additional 17 h under the same conditions. Cells without TNFα antagonists and complement serum treatment served as controls. The plate was then read at 530/590 nm excitation/emissions on a SpectraMax M2^e^ microplate reader. For data analysis, cell death was calculated as follows: cell death (%) = (Value _control_ − Value _drug_) / Value _control_ ×100. The dose-response curve was fitted with a 4-parameter model using GraphPad Prism 5 software. Rituximab was also tested against WIL2-S (CD20+) cells as a system positive control and tested against mTNFα-expressing CHO cells as a system negative control.

### Apoptosis assay

mTNF-transfected Jurkat T cells were untreated or stimulated for 24 hours with 100 nM TNFα antagonists or Rituximab. The cells were stained with FITC Annexin V Apoptosis Detection Kit (BD Biosciences) for 15 minutes in a dark room at room temperature. Apoptosis was measured by FACSCalibur flow cytometer for Annexin V positive cells.

## Results

### T0001 exhibited the strongest neutralizing activity on sTNFα induced cytotoxicity

**Fig 1:**
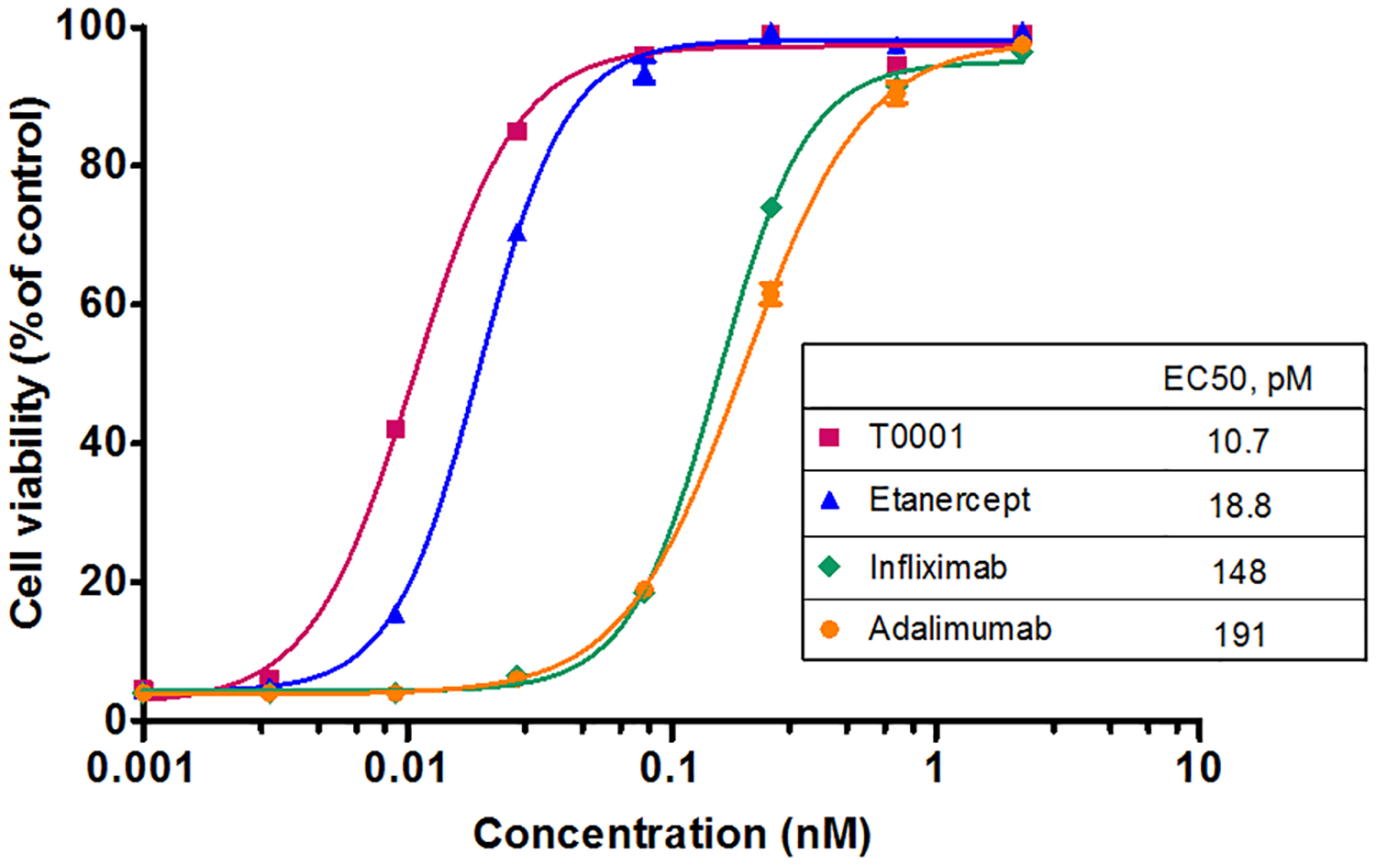
The neutralizing activity of TNFα antagonists on sTNFα-induced cytotoxicity in mouse L929 cells. 1.5×10^4^ L929 cells treated with TNFα antagonists in presence of 1 μg/ml actinomycin D and 20 IU/ml sTNFα for 18 h at 37°C, followed by MTS test. The cell viability expressed as the percentage of the control group as described in “Materials and methods”.

We have previously shown that the equilibrium dissociation constant of T0001 to sTNFα was 3.7-fold higher than that of Etanercept [4], whereas another study reported that etanercept has a 2.6-fold greater affinity to sTNFα than that for adalimumab [13]. Here, we compared the neutralizing activity of T0001 with etanercept and mAbs on sTNFα-induced cytotoxicity in murine fibrosarcoma L929 cells. The L929 cell line is a well-established cellular model to study necroptosis induced by sTNFα [19]. The calculated EC_50_ are shown in Fig 1; two TNFR-Fc fusion proteins exhibited approximately ten times stronger neutralizing activity than two mAbs, whereas T0001 showed nearly double the activity to etanercept.

### T0001 showed similar binding activity on mTNFα with mAbs

**Fig 2:**
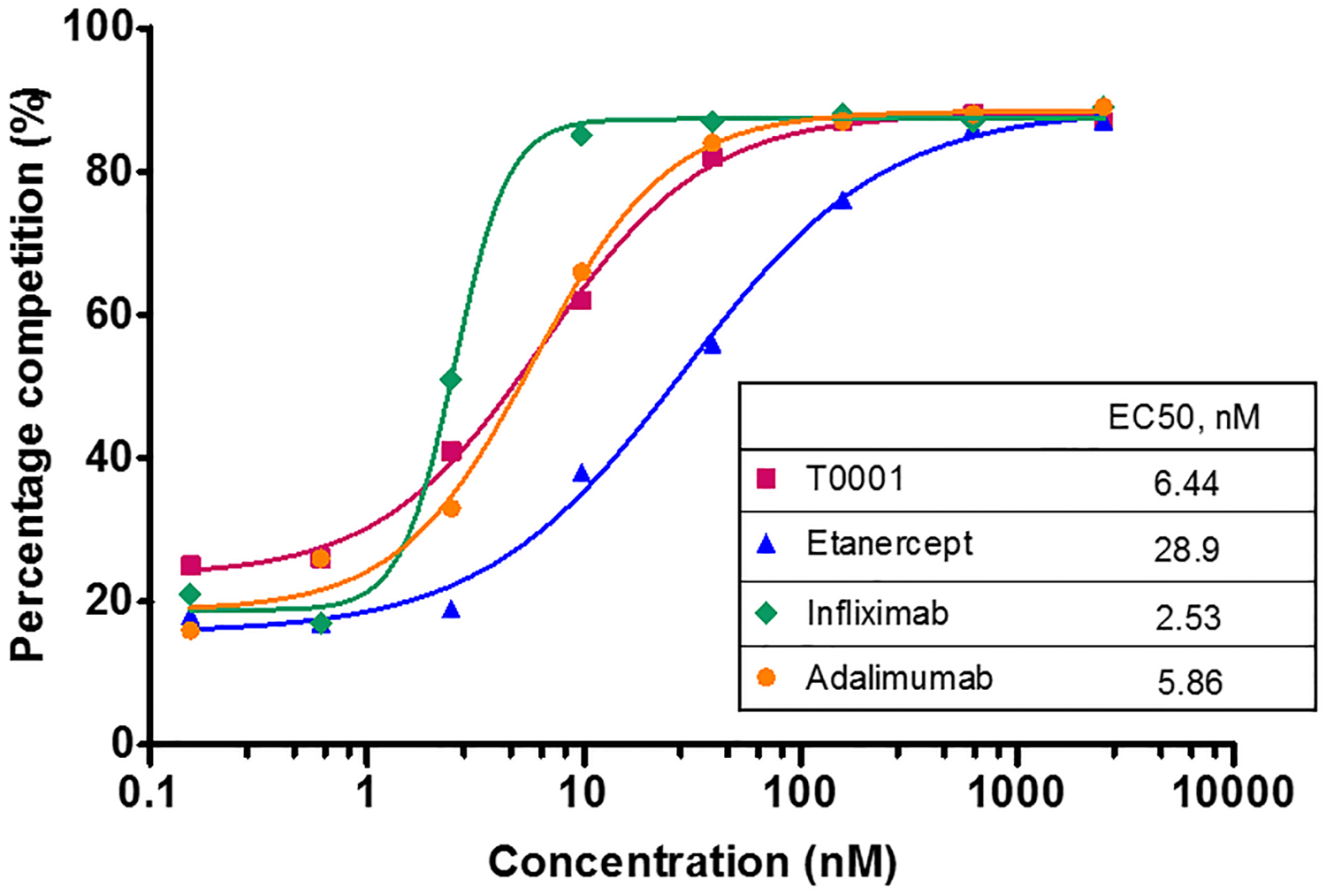
Competitive binding of TNFα antagonists to mTNFα on transfected cells. 5×10^5^ mTNF expressing CHO cells were incubated with TNFα antagonists competed with 13.3 nM biotin-T0001 for 0.5 h at 4°C, followed by incubation with R-phycoerythrin-conjugated avidin as a secondary antibody for 0.5 h at 4°C. The binding of TNFα antagonists to mTNFα expressed as the percentage competition as described in “Materials and methods”.

As the basis of Fc effector functions, we first studied the binding activity of T0001 compared with etanercept and mAbs to transmembrane TNFα. In the present study, competitive flow cytometry was used to assess the binding of TNFα antagonists to mTNFα expressing CHO cells. The biotin-T0001 achieved saturation binding to these cells at 40 nM. When competing with an EC_50_ concentration of biotin-T0001 (~13.3 nM), infliximab was the most competitive among the four TNFα antagonists (Fig 2). T0001 and adalimumab exhibited similar competitiveness; their binding activity to mTNF decreased by approximately 60% compared with infliximab. Etanercept had the poorest competitive binding ability.

### Binding of TNF antagonists to FcγRIIIa on transfected cells

**Fig 3:**
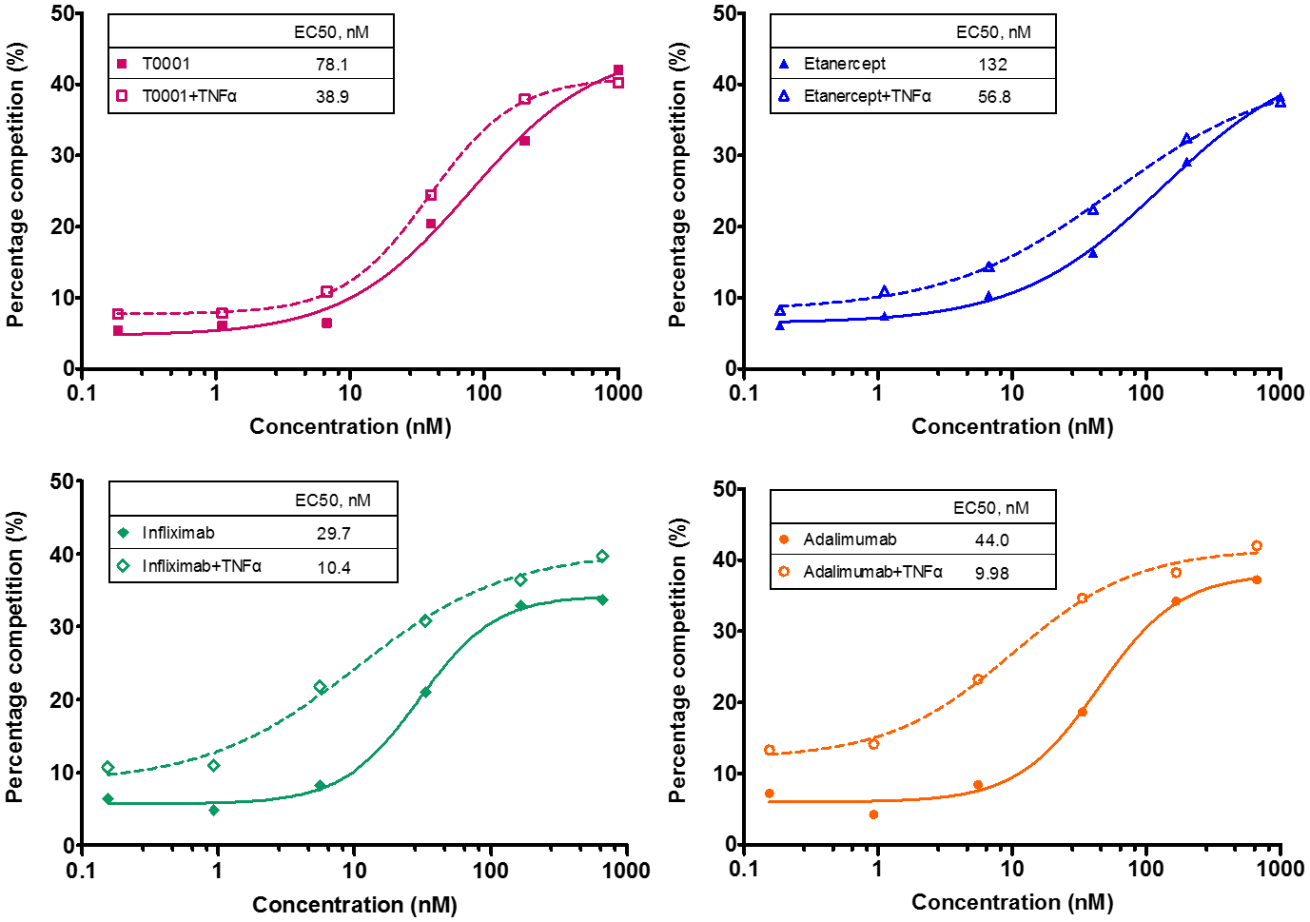
Competitive binding of TNFα antagonists to FcγRIIIa on transfected cells. 2×10^5^ Jurkat cells transfected with FcγRIIIa(158V) were incubated with TNFα antagonists competed with 6.7 nM biotin-IgG1 alone or in the presence of 1:1 molar excess sTNFα for 0.5 h at 4°C, followed by staining with Streptavidin R-PE Conjugate as a secondary antibody for 0.5 h at 4°C. The binding of TNFα antagonists to FcγRIIIa expressed as the percentage competition as described in “Materials and methods”.

The Fc portion of IgG is known to bind Fc receptors. All four TNFα antagonists tested here bear the Fc portion of human IgG1. Activation through FcγRIIIa on NK cells can result in the triggering of lytic mechanisms by ADCC. Flow cytometry was used to assess the binding of TNFα antagonists to FcγRIIIa (158V) expressing Jurkat cells through competition with biotin-IgG1. In the absence of TNFα (solid lines), two mAbs exhibited better competitive binding to FcγRIIIa than the Fc-Fusion proteins (Fig 3). In the presence of TNFα, there was a 4-fold increase in competitive binding by the mAbs, whereas there was only a 2-fold increase in competitive binding observed for T0001 or etanercept. Among the tested drugs, infliximab showed the best potential for binding to FcγRIIIa.

### T0001 possessed potential ADCC activity in a reporter gene assay and a PBMC-based assay

ADCC assays are commonly performed using peripheral blood mononuclear cells (PBMCs), natural killer (NK) cells or engineered cell lines as effector cells. In this study, a reporter gene assay (RGA) and a PBMC-based assay were used to assess the ADCC of TNF antagonists. The mTNFα-expressing CHO cells were used as target cells, while an engineered reporter cell line expressing FcγRIIIa (158V) and luciferase reporter gene, under the control of the NFAT promoter and PBMCs were used as effector cells, respectively.

**Fig 4:**
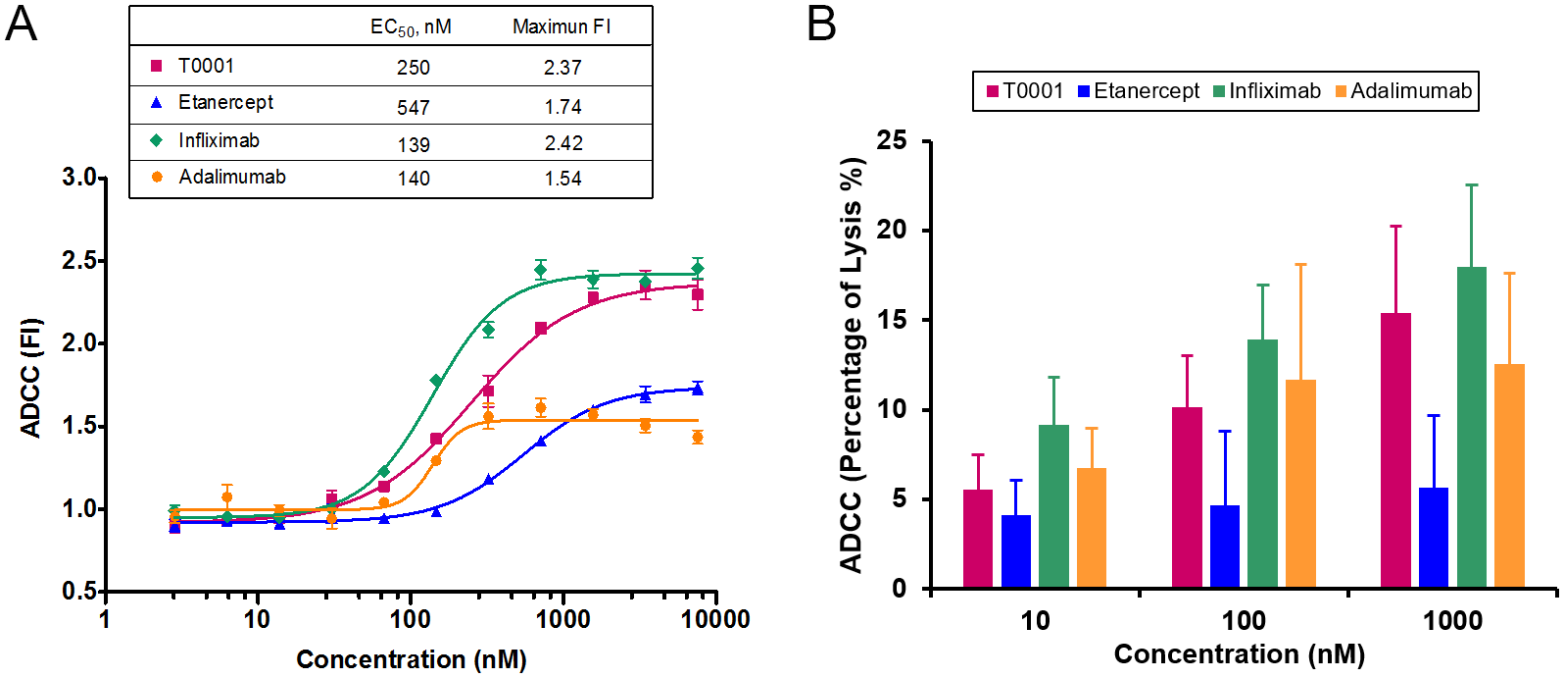
Ability of TNFα antagonists to mediate ADCC. Jurkat-NFAT luciferase reporter cells (A) or PBMCs (B) were used as effector cells and mTNF-expressing CHO cells were used as targets (E:T ratio 20:1) in ADCC assays. TNFα antagonists were serially diluted and incubated with the CHO cells, followed by incubation with the effector cells for 6 h at 37°C. In report gene assay, ADCC activity was measured for luciferase production using a luminescent substrate. ADCC expressed as the fold of induction (FI). In PBMC-based assay, ADCC activity was measured for LDH release and expressed as percentage of lysis.

In RGA, cross-linking of FcγRIIIa on the reporter cells by antibodies and Fc-Fusion proteins bound to target cells leads to the activation of the NFAT response element and expression of luciferase. The potency (EC_50_) and reporter response (maximum FI) were used as the assay outputs to compare the ADCC activity of TNF antagonists. At the E:T ratio of 20:1, infliximab exhibited minimum EC_50_ and highest maximum FI values, indicating stronger ADCC reporter activity than the other three TNF antagonists (Fig 4A). As expected, the two mAbs showed higher potency than the two Fc-fusion proteins. In the group of Fc-Fusion proteins, T0001 showed more than double the ADCC potency of etanercept, which may be attributed to its stronger binding to the target cell. Interestingly, the reporter response of T0001 was higher than adalimumab and similar to infliximab, which indicated T0001 might act as a Class II antibody in ADCC function. Using a reporter gene assay, the Class I control antibody, Rituximab, was tested as a system control. At the optimized E:T ratio of 1:3 for Rituximab, it detected no luciferase signal on mTNFα expressing CHO cells, while using WIL2-S (CD20+) cells as target cells, the dose-response curves of Rituximab showed a significantly high ADCC activity (EC_50_ was 0.14 nM, Maximum FI was 18).

Classical PBMC-based assay were performed using LDH release as readout to validate ADCC detected by RGA. T0001 showed similar potency in PBMC-based assay (Fig 4B), confirming the critical role of T0001 in ADCC effector function. This result also demonstrated that the RGA possessed the capability to detect ADCC function that is biologically relevant.

### Binding of TNF antagonists to C1q

**Fig 5:**
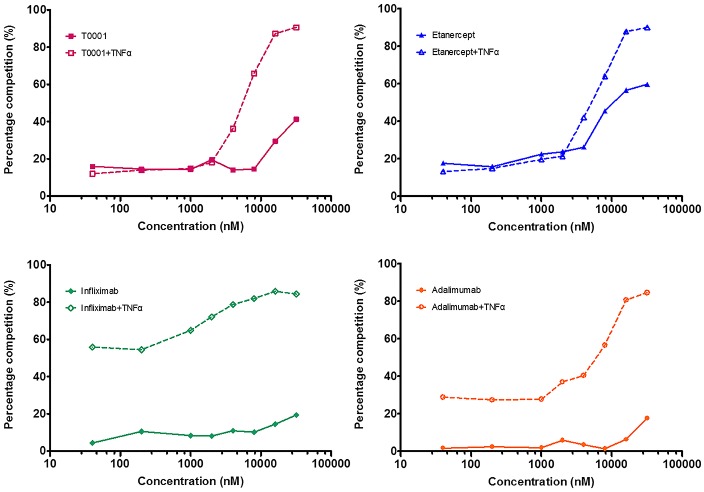
Competitive binding of TNFα antagonists to C1q. 96-well plates were coated with 5 μg/mL goat anti-human C1q antibody for 16 h at 4°C, and 3 μ/mL C1q was added to each well and incubated for 2 h at RT. 133.3 nM biotin-IgG1 and varying concentrations of TNFα antagonists were added to the plates alone or in the presence of a 0.8-fold molar excess of sTNFα, incubated at RT for 15 h and then followed by staining with streptavidin-HRP as a secondary antibody for 1 h at RT. The binding of TNFα antagonists to C1q expressed as the percentage competition as described in “Materials and methods”.

Antibodies are capable of initiating the classical complement pathway upon binding to their specific cell surface antigen. The ability of TNFα antagonists to bind with the complement component C1q was assessed using plate-based assays competition with biotin-IgG1. All of the four agents showed poor competitive binding to C1q in the absence of exogenous sTNFα. However, in the presence of sTNFα, mAbs, but not T0001 or etanercept, shows markedly competitive binding to C1q (Fig 5). Infliximab achieved the maximum improvement among the agents, but the TNFR-Fc Fusion proteins did not gain their competition shift until 4000 nM.

### T0001 mediated higher CDC activity than etanercept but mAbs

**Fig 6:**
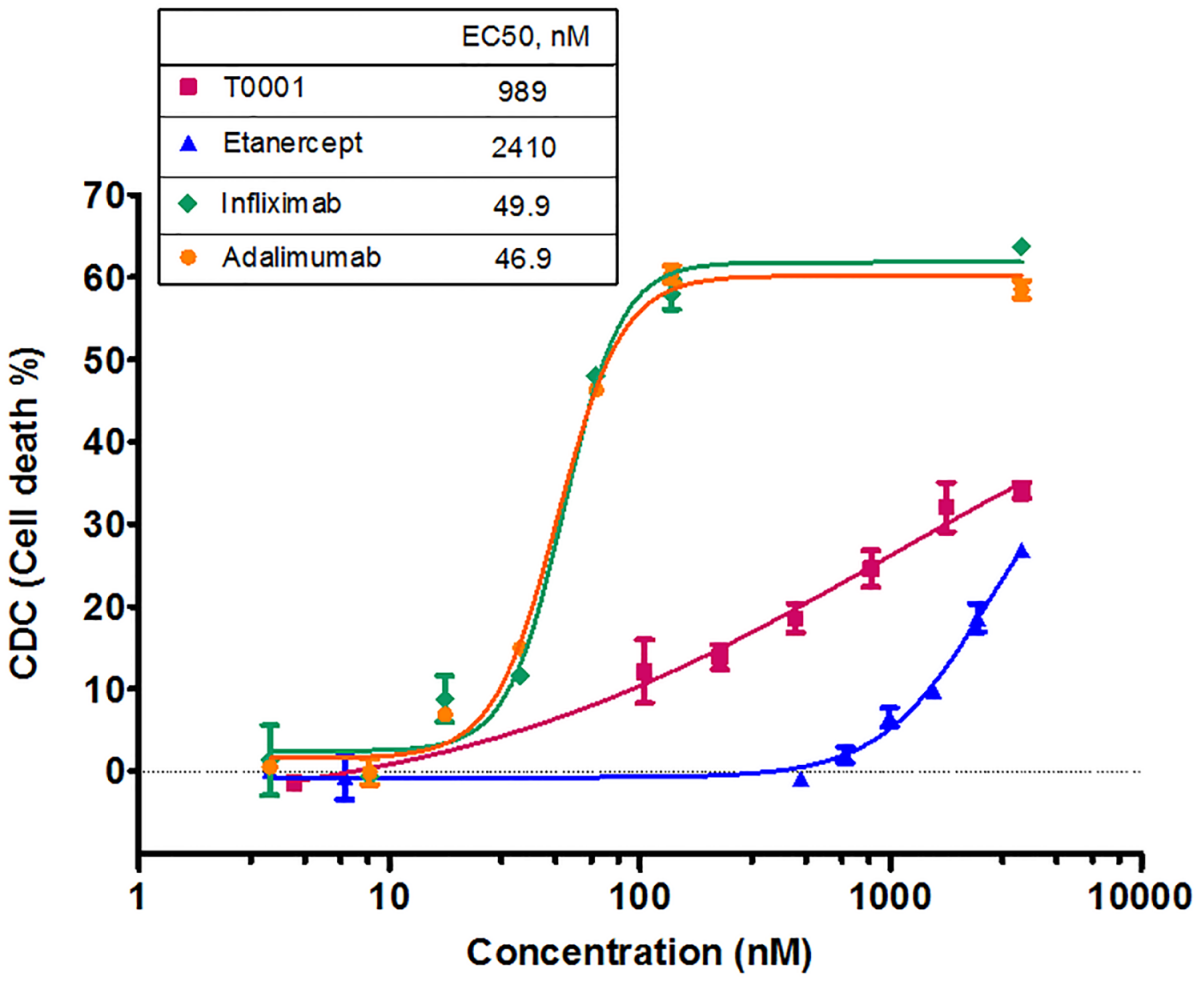
Ability of TNFα antagonists to mediate CDC. 5×10^4^ mTNF-expressing CHO cells were incubated with TNFα antagonists for 1 h at 4°C, followed by incubation with 8% human complement serum for 2 h at 37°C. At the end of incubation, AlamaBlue dye was added into each well and the plate was incubated for additional 17 h before read at 530 nm excitation/590 nm emission on a fluorescence plate reader. CDC expressed as the percentage of cell death as described in “Materials and methods”.

Cell death can be mediated by the complement component, C1q. To assess the degree of cell death associated with these agents, we used the mTNFα-expressing CHO cells as target cells incubated in the presence of TNFα antagonists. Cell death via CDC was observed in a dose-dependent manner when all of the agents were added to mTNFα-expressing CHO cells in the presence of a human complement (Fig 6). However, the mAbs were far more potent at inducing CDC than T0001 and etanercept in this assay. In the group of Fc-Fusion proteins, the cell death was below 40%, even for very high doses of the drugs. Despite low CDC activity, T0001 was calculated to have a 2.4-fold higher CDC potency than etanercept, which may be explained by its stronger binding affinity to the target cell. The Class I control antibody, Rituximab, was tested as a system control. Rituximab detected no CDC on mTNFα-expressing CHO cells. However, using WIL2-S (CD20+) cells as target cells, the dose-response curves of Rituximab showed a significantly high CDC activity (EC_50_ was 2.8 nM, Maximum cell death was 85%).

### T0001 can induce apoptosis on mTNF-transfected Jurkat cells

**Fig 7:**
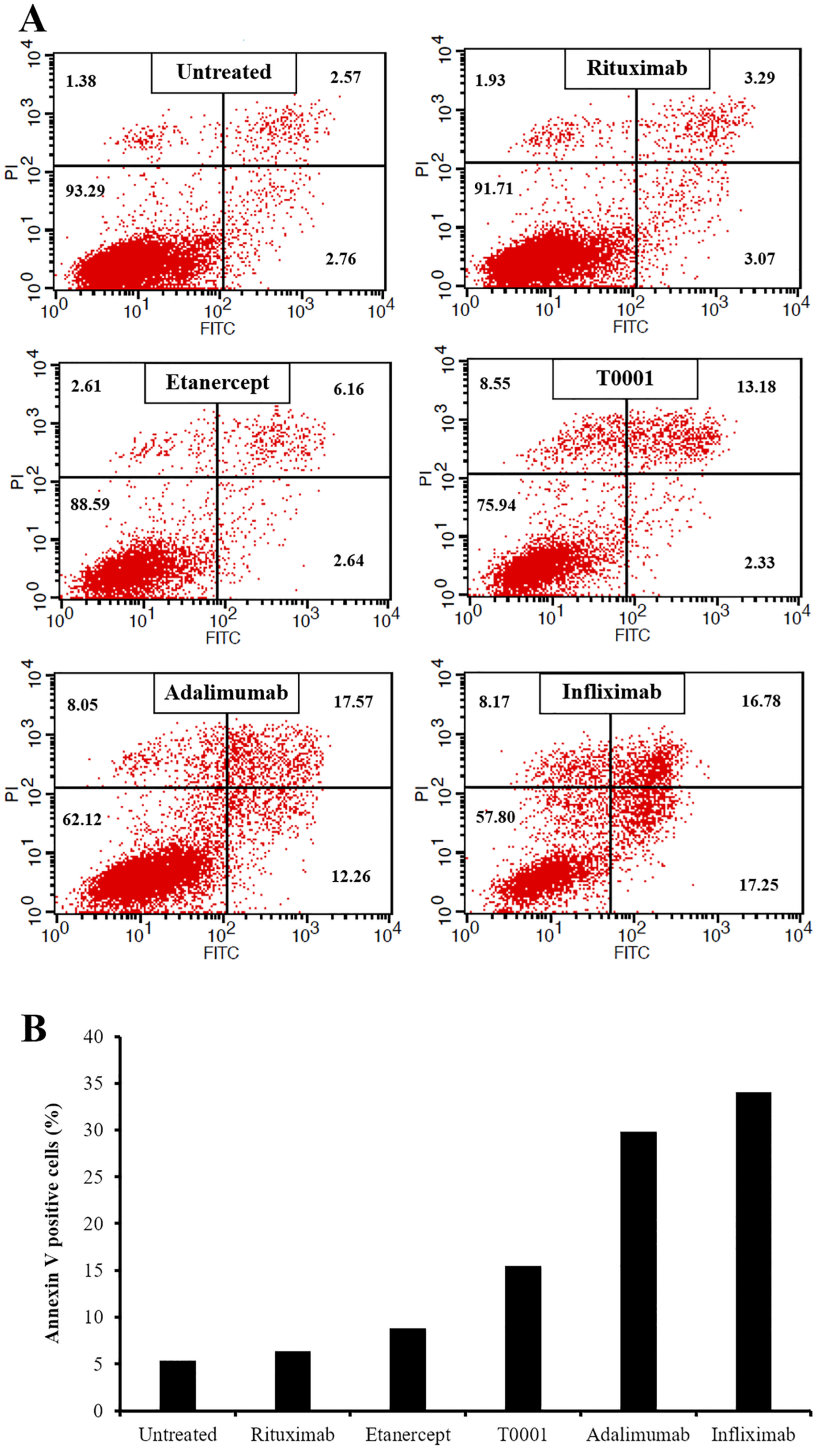
Apoptosis induced by TNFα antagonists in mTNF-transfected Jurkat T cells. (A) mTNF-transfected Jurkat T cells were untreated or treated with TNFα antagonists or rituximab, at 100 nM and cultured for 24 hours. The stimulated cells were stained with FITC-conjugated Annexin V and PI and were then analyzed by flow cytometry. Ten thousand cells were measured and plotted. The proportion of cells residing in each quadrant is expressed as a percentage. (B) The proportions of Annexin V-positive cells in each sample after stimulation are indicated as solid columns.

The outside-to-inside (reverse) signaling through mTNF is consider the essential MOA of anti-TNF agents to control IBD. To explore the reverse signal induced by T0001, we investigated apoptosis induction. mTNF-transfected Jurkat T cells were stimulated for 24 hours with TNFα antagonists or rituximab at 100 nM. Stimulation with T0001 increased the number of Annexin V-positive cells to 15.51%, which means T0001 could induce apoptosis on mTNF-transfected Jurkat cells. Meanwhile, etanercept had almost no effect on apoptosis, and infliximab and adalimumab induced stronger apoptosis than T0001. (Fig 7).

## Discussion

**Fig 8:**
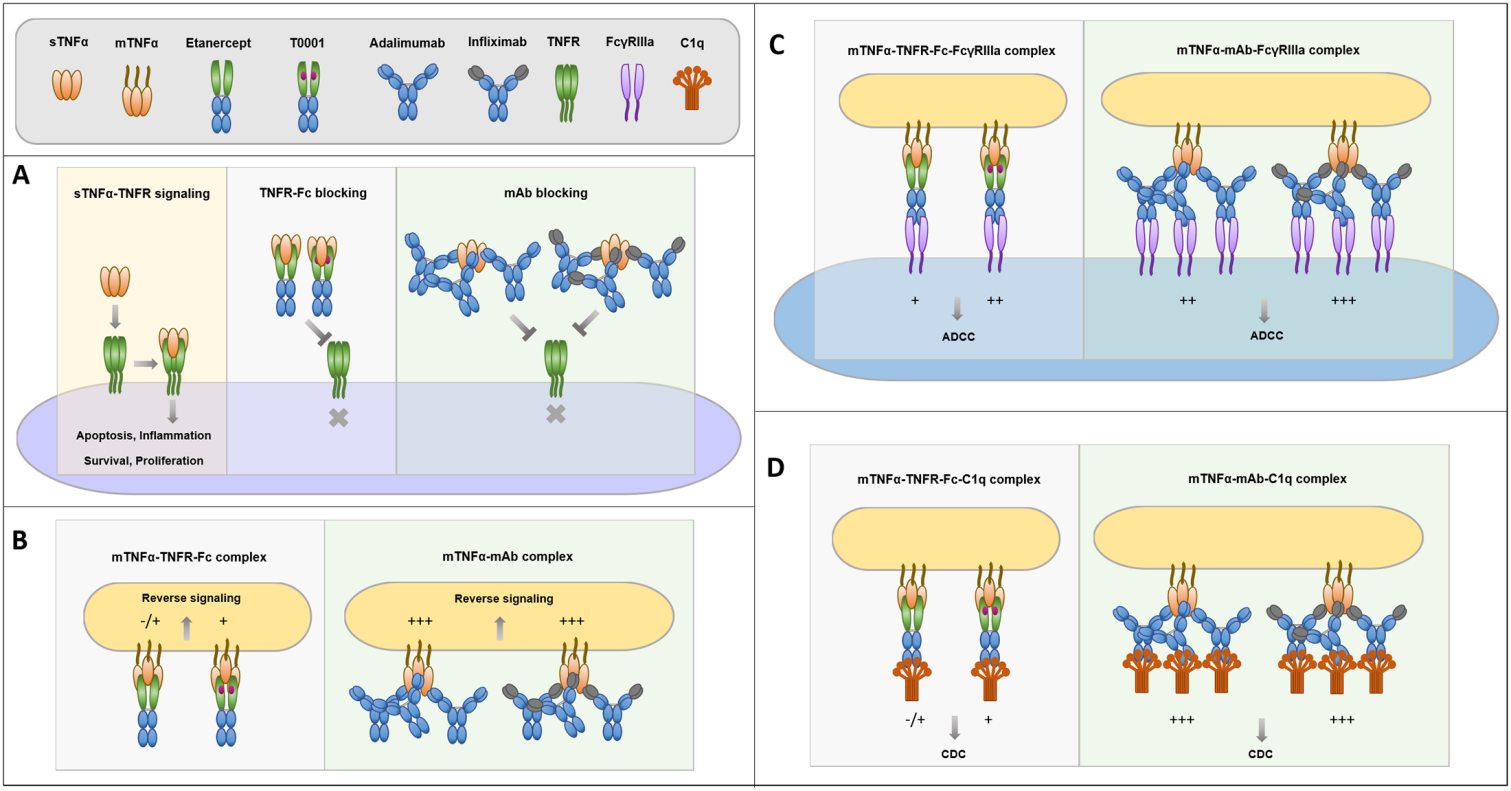
Schematic illustrations summarizing the different actions between TNFR-Fc fusion proteins and monoclonal antibodies. TNFR-Fc fusion protein (T0001 or etanercept) binds TNFα in a 1:1 stoichiometry, while up to three molecules of mAb (adalimumab or infliximab) can bind to each sTNFα trimer. (**A**) When blocking sTNFα to recruit TNFR trimer, one TNFR-Fc fusion protein molecule is sufficient, but at least two mAb molecules may be needed. (**B**) When inducing reverse signal though mTNFα, mAb formed more stable complexes with mTNFα and exhibited higher avidity than TNFR-Fc fusion protein. (**C**) When activating ADCC, TNFR-Fc fusion protein binds both mTNFα trimer and FcγRIIIa in a 1:1:1 stoichiometry, while three mAb molecules can bind to each mTNFα trimer, consequently recruiting three FcγRIIIa. (**D**) When activating CDC, TNFR-Fc fusion protein binds both mTNFα trimer and C1q in a 1:1:1 stoichiometry, while three mAb molecules can bind to each mTNFα trimer, consequently recruiting three C1q.

Evidence from the neutralization of circulating sTNFα and reduced inflammation suggests that the MOA of TNFα antagonists are similar in RA. In this situation, TNFα antagonists prevent sTNFα from recruiting the TNFR trimer. The results of this study showed that the TNFR-Fc fusion proteins exhibited significantly stronger neutralization of sTNFα than mAbs. This is because one TNFR-Fc fusion protein monomer (bearing two TNFR molecules) can block a sTNFα trimer to TNFR, but two or three mAb monomers may be needed to effectively interfere with the association (Fig 8A). In the L929 cytotoxicity model, T0001 showed further improved activity than etanercept, which can be attributed to its higher affinity to sTNFα. In fact, TNFR-Fc can bind equally well to both TNFα and lymphotoxinα (LTα), whereas mAbs cannot. The actual potency of the two kinds of TNFα antagonists is identical in RA.

In contrast, mTNFα not only acts as a ligand but also can be activated to mediate reverse signaling into cells expressing this molecule. Previous data demonstrated that biologic effects-namely apoptosis, cell cycle arrest, and IL-10 production-are mediated by reverse signaling through mTNFα only by infliximab, which might explain, at least in part, the difference in clinical effects between infliximab and etanercept in CD [20]. Here, we assessed the Fab and TNFR-mediated reverse signaling of mAbs and TNFR-Fc fusion proteins through engagement of mTNFα on the target mTNFα-transfected Jurkat T cells and transmission of a signal into the cell which cause apoptosis. Relevant to mTNF binding ability, infliximab induced strongest apoptosis, considering that infliximab formed most stable complexes with mTNFα on transfected cells with highest avidity (Fig 8B). T0001 also induced apoptosis signal, although the induced signal intensity was only half that of adalimumab or infliximab induced. Etanercept bound to mTNF on Jurkat T cells resulted in a barely detectable apoptosis signal. This result indicated that T0001 may have potential in IBD through increased binding to mTNFα and the resultant improved reversed signaling.

In addition, the Fc region of anti-TNFα mAbs may be involved in other potential mechanisms, such as ADCC and CDC. The differences in clinical activity and indication for use among TNFα antagonists illustrate that anti-TNFα mAbs but not etanercept is suitable for CD and UC [3, 15, 21]. Health Canada stated that “ADCC may be an active mechanism of action for infliximab in the setting of IBD, but not in the setting of rheumatic disease;” however, differences in the ability of infliximab and its biosimilar CT-P13 to induce ADCC could not be ruled out [22]. Therefore, a study of Fc effector functions of the high-affinity variant of TNFR-Fc may explore its clinical indication in the future. In the crosslinking models of ADCC and CDC, an antibody first binds to its antigen on target cells, after which the Fc portion is recognized by FcγR on the effector cells or the C1q component. In this study, we studied the binding of the TNFα antagonists to the membrane-bound TNFα and binding to FcγRIIIa and C1q.

A previous study of complexes indicated that one or two etanercept monomers are bound to a TNFα trimer. Adalimumab and infliximab can bind to both monomer and trimer forms of TNFα to form a variety of complexes. Therefore, more mAb molecules bind to mTNFα with higher avidity than TNFR-Fc. Here, we evaluated whether the binding of TNFα antagonists competed with biotin-T0001 to mTNFα using a flow cytometry with a cell line (CHO) that had been transfected with a form of mTNFα that resists cleavage by TACE. A non-radioactive and competitive method was used here to avoid differences in label efficiency and for the ease of handling in a common lab. As expected, both mAbs and T0001 showed higher binding activity than etanercept. A slower dissociation speed than that of etanercept is critical for its stronger binding to mTNFα [4], which helps T0001 to form more stable complexes with mTNFα.

Next, we evaluated the binding of TNFα antagonists competing with non-TNFα targeted biotin-IgG1 to FcγRIIIa and C1q by flow cytometry and plate-based assays, respectively. FcγRIIIa receptor has a low affinity for Fc and only binds to aggregated Fc or antibodies complexed with multivalent antigens with high avidity. When TNFα antagonists competed for binding to FcγRIIIa (158V)-expressing Jurkat T cells, binding by the anti-TNF antibodies, but not by the TNFR-Fc fusion proteins, was significantly enhanced in the presence of TNFα. This increased binding was likely due to the formation of large complexes by the mAbs. Both mAbs and TNFR-Fc fusion proteins demonstrated low-level binding to C1q in the absence of exogenous TNFα, which was consistent with the study of Arora et al [16]. However, upon the addition of TNFα, the mAbs, but not TNFR-Fc fusion proteins, showed considerably increased binding to C1q. The large protein complexes formed by the mAbs may behave similarly to aggregated Ig, and the binding of these agents to C1q may reflect the ability of multiple Ig molecules in inducing CDC.

In the light of these binding studies, we evaluated the Fc effector functions *in vitro*. Contrary to previous studies of ADCC by TNF antagonists [11,12,23], we developed a reporter-based ADCC assay that used an engineered Jurkat stable cell line as the source of effector cells instead of a classic PBMC-based ADCC assay. The ADCC reporter assay measured the Fc effector function in a reproducible and quantitative manner. All of the four anti-TNF agents exerted ADCC activities. Adalimumab and infliximab showed 4-fold more potency than etanercept. This could be explained by the additive effects of the stronger affinity to mTNFα and more molecules bound to the mTNFα trimer (Fig 8C). The potency of T0001 was comparable to mAbs, and its reporter response was higher than that of adalimumab. These results indicated that T0001 has a potent ADCC activity on account of its higher avidity to mTNFα. Nevertheless, the mAbs were far more potential than the TNFR-Fc fusion proteins in CDC. The dramatically increased binding to C1q of mAbs indicated that only the multiple Fc regions of Ig molecules own the potential to trigger the classical complement pathway (Fig 8D). Since ADCC is a more important MOA than CDC in IBD, T0001 may be used for treating UC and CD.

There are several therapies available for IBD, depending on the stage, location and behavior of these diseases. Despite the immunological differences between UC and CD, TNFα is hypothesized to play a key role in regulating the pathogenesis of both diseases [23]. Notwithstanding the high cost involved, the anti-TNFα mAbs show a high index of remission, enabling a significant reduction in the cases of surgery and hospitalization [24]. In RA, patients receiving adalimumab or infliximab had significantly higher rates of dose escalation than patients receiving etanercept, and the related costs were also higher [25]. Therefore, improving Fc effector functions by increasing mTNFα binding characteristics may allow a patient who is non-responsive to etanercept to respond to T0001 and subsequently benefit from this new drug at a lower total cost of care.

In conclusion, the present study suggests that T0001 may not only exert improved efficacy in treating RA because of its higher affinity to sTNFα but also have a therapeutic potential in CD and UC due to its increased avidity and more stable binding to mTNFα, resulting in improved Fc-associated functions, especially ADCC.
